# A Review of Data Analytic Applications in Road Traffic Safety. Part 1: Descriptive and Predictive Modeling

**DOI:** 10.3390/s20041107

**Published:** 2020-02-18

**Authors:** Amir Mehdizadeh, Miao Cai, Qiong Hu, Mohammad Ali Alamdar Yazdi, Nasrin Mohabbati-Kalejahi, Alexander Vinel, Steven E. Rigdon, Karen C. Davis, Fadel M. Megahed

**Affiliations:** 1 Department of Industrial and Systems Engineering, Auburn University, Auburn, AL 36849, USA; azm0127@auburn.edu (A.M.); qzh0011@auburn.edu (Q.H.); alexander.vinel@auburn.edu (A.V.); 2 College for Public Health and Social Justice, Saint Louis University, St. Louis, MO 63103, USA; miao.cai@slu.edu (M.C); steve.rigdon@slu.edu (S.E.R.); 3 Carey Business School, Johns Hopkins University, Baltimore, MD 21202, USA; yazdi@jhu.edu; 4 Jack H. Brown College of Business and Public Administration, California State University at San Bernardino, San Bernardino, CA 92407, USA; nasrin.mohabbati@csusb.edu; 5 Department of Computer Science and Software Engineering, Miami University, Oxford, OH 45056, USA; karen.davis@miamioh.edu; 6 Farmer School of Business, Miami University, Oxford, OH 45056, USA

**Keywords:** crash risk modeling, data visualization, descriptive analytics, highway safety, predictive analytics

## Abstract

This part of the review aims to reduce the start-up burden of data collection and descriptive analytics for statistical modeling and route optimization of risk associated with motor vehicles. From a data-driven bibliometric analysis, we show that the literature is divided into two disparate research streams: (a) predictive or explanatory models that attempt to understand and quantify crash risk based on different driving conditions, and (b) optimization techniques that focus on minimizing crash risk through route/path-selection and rest-break scheduling. Translation of research outcomes between these two streams is limited. To overcome this issue, we present publicly available high-quality data sources (different study designs, outcome variables, and predictor variables) and descriptive analytic techniques (data summarization, visualization, and dimension reduction) that can be used to achieve safer-routing and provide code to facilitate data collection/exploration by practitioners/researchers. Then, we review the statistical and machine learning models used for crash risk modeling. We show that (near) real-time crash risk is rarely considered, which might explain why the optimization models (reviewed in Part 2) have not capitalized on the research outcomes from the first stream.

## 1. Introduction

Despite the significant technological advances in motor vehicle sensing technologies (e.g., lane departure detection and collision mitigation sensing systems), road crashes have remained a pressing global health issue. The World Health Organization estimated that road injuries are the 8th leading cause of death worldwide, resulting in 1.4 million deaths annually [1]. Perhaps more importantly, the incidence of such crashes and their severity are on the rise. By 2030, traffic-related deaths are predicted to become the 7th leading cause of death worldwide [1]. The increase in annual deaths is seen in low- and high-income countries alike. For example, in the U.S., an estimated 37,133 people died in road crashes in 2017 [2], which constituted a 7.5% increase from the average annual deaths recorded in 2012–2016 [3]. In addition to the massive loss of life, motor vehicle (which is used to capture passenger cars, motorcycles, buses and trucks) crashes cause significant economic losses. According to the World Health Organization [4], “road traffic crashes cost most countries 3% of their gross domestic product.” In the U.S., it is estimated that the total value of societal harm from motor vehicle crashes exceeds $830 billion annually [5], which is equivalent to ≈4.4% of the country’s gross domestic product [6].

Consequently, there are multiple diverse streams of research dedicated to curbing such driving-related risks. This review focuses on data analytics approaches, which revolve around the idea of using data to characterize and predict traffic risk in order to prescribe better (safer) routes, driver assignments, rest breaks, etc. With the advances in information technology it is possible to collect ever increasing amounts of relevant data, such as comprehensive incident databases, real-time driving data feeds, or relevant factor characteristics (e.g., detailed historical and forecasted weather and traffic reports). Further, there has been a tremendous improvement in the variety and capabilities of data analytics tools and methods that can be applied to all steps of modeling (data collection, processing, prediction, or prescription). The goal of this study then, is to pull together and categorize the existing literature on different aspects of research relevant to enabling data-driven analytics approaches to traffic safety.

The study was inspired by an observation that there exists an apparent disconnect between two essential facets of pertinent research efforts: statistical modeling of crash risk on one hand and prescriptive modeling for decision making on the other. For example, it is very common in operations research (OR) literature to assume that the crash probability is time-invariant [7,8], and is, in fact, in the range of $10^{-8}$ to $10^{-6}$ per mile [9]. This contradicts the findings from the predictive stream of research, with multiple efforts studying the effect of real-time crash risk factors (traffic and weather conditions) on the likelihood of a crash. According to the reviews in [10,11] different traffic and weather conditions would result in different crash risk profiles, bringing into question the effectiveness of the methods often used by OR community for considering risk in decision-making process.

**Figure 1:**
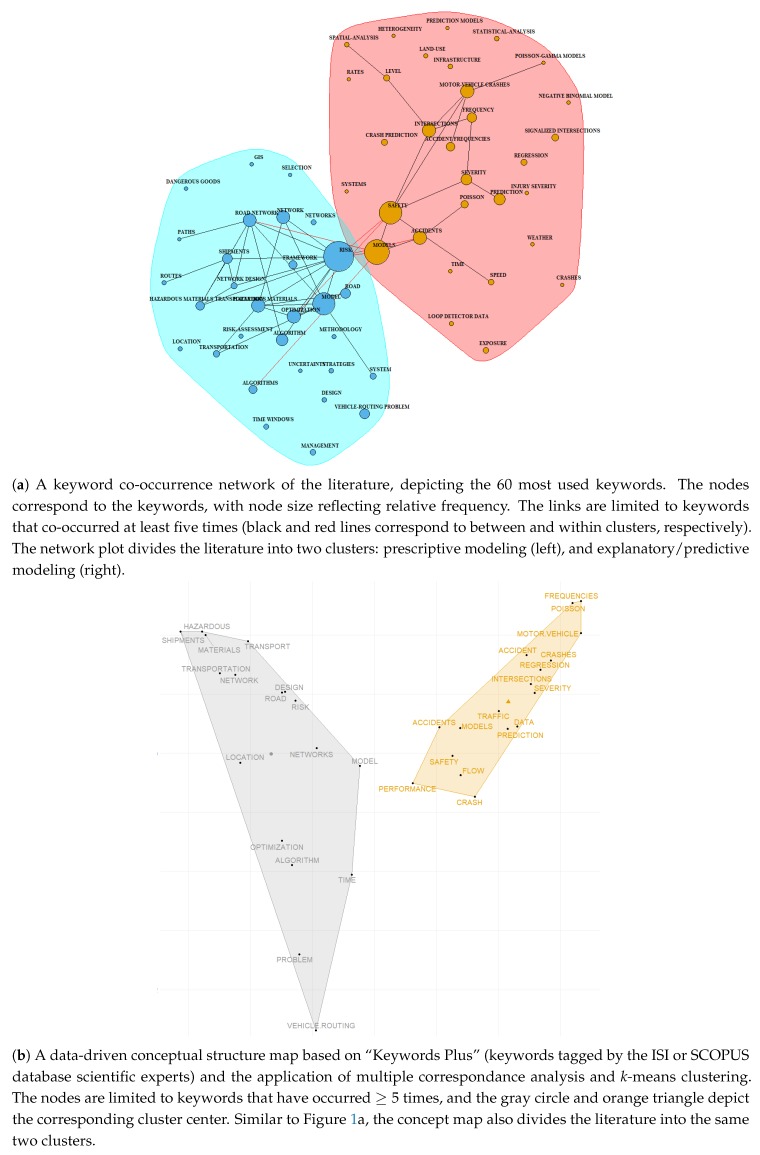
A bibliographic analysis of the literature using the *bibliometrix* package in **R**.

In order to further examine this apparent gap we have conducted a more formal bibliographic study. Based on the keywords and search strategy described in the Supplementary Materials Section, we identified 856 relevant documents (i.e., published articles, proceeding papers, and book chapters). To categorize these documents for this review, a text/bibliometric analysis was performed using the *bibliometrix* **R** package [12], with the goals of: (a) examining the co-occurrences of keywords within documents since this shows a link between the topics captured by these keywords; and (b) constructing a conceptual structure map of the literature based on a more streamlined keywords list (“Keyword Plus”, see [13] for a detailed introduction). The results are shown in Figure 1a,b, respectively.

In the keyword co-occurence network, induced by the documents found, a pair of keywords is connected by a link, if they appear in the same document (the links are weighted according to the number of co-occurrences). This network is then clustered with K-means clustering algorithm (all parameters selected automatically by *bibliometrix* package). The clusters and most important links (corresponding to more than four co-occurrences) are depicted on Figure 1a with the black and red links depicting within-cluster and between-cluster connections respectively. The conceptual structure map (Figure 1b) aims at identifying the common emerging concepts in the expanded “Keyword Plus” network. Here, dimensionality reduction technique (multidimensional scaling) is applied to the concept co-occurrence network in order to project it to two dimensions, and the result then clustered with a K-means clustering algorithm. More details on the precise implementation can be found in [12].

Based on Figure 1, two important observations can be made. First, the literature can indeed be grouped into two main groups: (a) an explanatory/predictive modeling stream, where the keywords emphasize the collected data (loop detector data), predictors (traffic, weather, time and/or infrastructure), models used (regression, spatial-analysis, Poisson-gamma and negative binomial), and model outcomes (rates, crash frequencies, and crash prediction); and (b) a prescriptive modeling stream, where the focus is on developing algorithms to manage risk, particularly for hazardous materials (hazmat) trucking, through the selection of paths and routes. Second, the cluster agreement between the keyword co-occurrence network and the concept map generated using the Web of Science’s Keywords Plus field implies that there is a clear division between the two research streams, despite the fact that the outputs from the first stream should be inputs for the optimization models used for prescriptive decision-making. Based on the second insight and a separate thorough examination of the relevant operations research (OR) literature we can then conclude that the prescriptive literature largely ignores the recent results on factors influencing crash risk.

Against this backdrop, the primary purpose of this review is to help bridge the gap between the different research streams that relate to the modeling and minimization of crash risk. Our goal is to bring the research into better focus and to encourage future work that crosses the siloed divisions within the literature. To achieve this goal, we divide this review into two parts. Part 1 covers the sensing, data acquisition, data exploration, and explanatory/predictive modeling, i.e., focuses on the first research stream. Part 2 reviews the prescriptive modeling component (i.e., second stream), provides a simple case study for how both streams can be integrated, and presents ideas for future research. Note that the research presented in Part 2 primarily targets hazardous materials (hazmat) trucking operations, where optimization models are used to minimize crash risk through path/route selection and/or rest-break scheduling, while meeting delivery requirements. On the other hand, in Part 1, the research relates to both commuters and commercial drivers since the unit of analysis is a “road segment”.

This paper is structured to follow the standard data analytics framework: data collection ⟶ data exploration ⟶ predictive modeling. The final part—prescriptive modeling—is discussed in Part 2 of this effort. We would like to emphasize that in addition to the need for connecting siloed research streams identified above, there also may exist a relatively high “start-up cost” for initiating new efforts in this area. Specifically, as we survey in the remainder of this paper, there exist multitudes of disparate datasets, data processing approaches and statistical methods that all may be relevant. Hence, the goal of this review is to attempt to reduce this burden by categorizing the existing efforts. The remainder of the first part of this review is structured utilizing a data analytic framework (data collection ⟶ data exploration ⟶ predictive modeling). We present an overview of the sensors and data collection mechanisms used in these studies in Section 2. In Section 3, we provide a taxonomy and review of the commonly utilized data exploration and summarization techniques. Then, we synthesize the explanatory/predictive modeling techniques used for crash risk modeling in Section 4. We offer our concluding remarks in Section 5, and provide links for our code and analysis in the Supplementary Materials Section.

## 2. Data Acquisition Protocols: An Overview of the Types of Collected Data and Their Associated Sensing Systems

In this section, we provide an overview of the data acquisition strategies typically used in motor vehicle safety studies as well as a brief to introduction to the corresponding sensing systems. The ability to extract such data is an indispensable component in any crash risk prediction study, yet it is typically under-described. Thus, we view this section as an important practical contribution of our review since a potential reason for the gap between the predictive and prescriptive analytic research streams can be attributed to the “large start-up burden”, associated with the lack of sufficient/targeted documentation for collecting quality data. While we primarily focus on U.S.-based systems, the protocols described here can be extended to many transportation locales. To facilitate and encourage the collection of data pertaining to important factor sets (per the reviews of Theofilatos and Yannis [10] and Roshandel et al. [11]) in future prescriptive studies, we provide **R** code that can be used to scrape data for many important crash risk predictors (see the link in our Supplementary Materials Section).

It must be emphasized that both data sources needed and data acquisition methods used to access these sources depend on the design of the study in question. Specifically, since this review is focused on the literature dedicated to models for quantifying crash risks, the corresponding studies can generally be divided into two main study designs: (a) retrospective case-control studies in which police crash reports are used, and (b) prospective naturalistic driving studies (NDS), in which a pre-specified set of drivers is followed for a certain period of time. As one can expect, the choice of study design affects the data collection mechanism (as well as the statistical methodologies used for analysis, which are discussed in Section 4). For the sake of completeness, we provide some background on each of these two design strategies in the following subsection.

### 2.1. Background: Study Designs

Most research on motor vehicle safety has assumed that the sampling unit is a spatiotemporal snapshot of a highway, i.e., researchers typically study a given section of a highway for a pre-specified time period. Note that it is not sufficient to study the conditions under which crashes tend to occur; one must also study the conditions under which crashes do not occur, and compare the two. The problem is analogous to that faced by epidemiologists when investigating the cause(s) of a disease, where they examine the prior behavior of individuals with and without a disease and attempt to identify differences in their prior behavior. The most common design that epidemiologists use is the case-control design. A number of individuals with the disease are first identified, representing the cases. The demographic and behavioral characteristics (e.g., age, sex, race, smoking status, body mass index, etc.) for the cases are then determined/computed. A control group, as similar as possible to the case group, is then identified. In a matched pair case-control study, each case is matched with one or more control subjects.

In motor vehicle highway safety applications, these retrospective case-control studies are typically conducted using police crash reports. In the U.S., crash reports include information pertaining to number of vehicles, involvement of pedestrians, number of injuries/fatalities, road type, crash location, date-time, intersection type, presence of a nearby work zone, weather conditions, and road surface conditions [14,15]. While a lot of information can be captured in these reports, case-control studies are inherently limited for two main reasons. First, the information captured in the crash reports combines: (a) factual information, e.g., type of road and number of vehicles involved in the crash; (b) information that is estimated by the police officer, e.g., classifying weather into one of pre-defined categories; and (c) information captured from witnesses which is subject to recall and/or information bias, e.g., it is often hard to gauge the veracity of information extracted from drivers involved in the crash. Second, the inference from case-control studies can be limited when the denominator (e.g., non-crashes or healthy individuals) is unknown to the researchers [16]. In highway safety research, traffic flows can be captured using cameras and on-the-road sensors; however, such information is not typically available for every road segment (e.g., in rural local roads and/or for all highway exits). Thus, this is a prevalent issue in existing case-control highway safety studies.

To alleviate the limitations in case-control studies, there has been an increasing number of prospective naturalistic driving studies (NDSs) in the past decade. Contrary to the case-control studies, the information is captured via one or more sensors that are mounted in the vehicle in an effort to collect [17]: (a) high-resolution real-time driving data under real-world circumstances; (b) location/GPS, speed, and multiple views of the driver/road; and (c) naturalistic/individualized driving behaviors that can help explain differences if a crash is observed during the study period. Compared to traditional case-control studies, NDSs resemble prospective cohort studies, where a pre-specified set of drivers is followed for a certain period of time. The sampling units here are the drivers instead of road segments, and all the events or non-events of the sample drivers are collected. Therefore, it is possible to compare the rates of events in NDSs. In addition, the data are automatically collected using sensors, which minimizes the impact of police/witnesses’ judgement in imputing the data and/or estimating values for certain predictors.

### 2.2. Outcome Variables Used in Crash Risk Modeling

**Figure 2:**
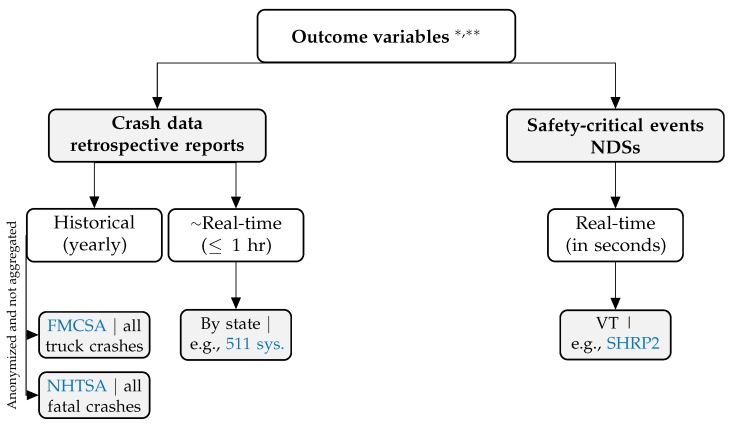
A hierarchical view of outcome variables in crash risk modeling studies. The first level captures the data type, the second level shows the frequency, and the third level highlights examples and sources. * Acronyms: FMCSA = Federal Motor Carrier Safety Administration, NHTSA = National Highway Traffic Safety Administration, VT = Virginia Tech. ** Code: To simplify the data collection process, we present the **R** code needed to scrape and clean these different data sources at: https://caimiao0714.github.io/TrafficSafetyReviewRmarkdown/.

In retrospective case-control studies, the most frequently used outcome variable is crash counts. In the U.S., historical crash data are hosted by different Department of Transportation (DoT) divisions depending on: (a) the types of vehicles involved, i.e., commercial vehicles or personal commuter vehicles; and (b) whether the crash resulted in any fatalities. When these models are utilized/deployed for predictive purposes, real-time traffic data can often be used as model inputs. In the U.S., such data can be obtained from state specific reporting systems. For example, the 511 reporting system highlighted in Figure 2, is the predominately used sensing system in the U.S. since it is used by more than 45 states [18]. On the other hand, in prospective NDSs, the use of safety-critical events (SCEs) as a proxy outcome variable is more common since: (a) NDSs do not focus on crash-prone highways, (b) SCEs have a much higher incidence rate than crashes, and (c) they are assumed to be positively correlated with the incidence of crashes [16,19]. SCEs are defined as events that avoid crashes by last-second evasive maneuver(s) [16]. The most commonly studied SCE is “hard brakes”, which can be detected using accelerometers/inertial measurement units mounted in the vehicle or through a driver’s smart phone. The identification of a “hard break” is threshold dependent; for example, several studies equate a “hard break” to a deceleration higher than 3.0 m/s${}^{2}$ [20,21]. Several detailed reviews have been published on surrogate indicators using in the field of traffic safety [22,23,24]. It is important to note that, while SCE has been extensively used as the outcome variable in NDSs, its validity and causal relationship with crashes have not yet been conclusively confirmed [25,26]. We provide a visual summary of the hierarchical nature of the described outcome variables in Figure 2.

### 2.3. Predictor Variables Used in Crash Risk Modeling

**Figure 3:**
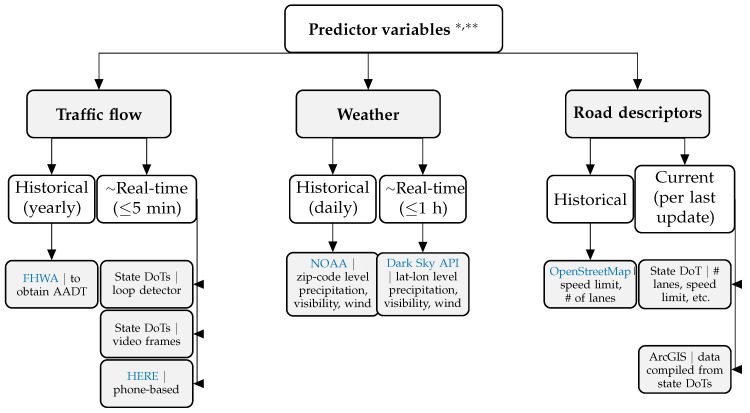
A hierarchy of predictor variables used in modeling crash risk. The first level captures the data type, the second level shows the frequency, and the third level highlights examples and sources. * Acronyms: AADT = Annual Average Daily Traffic, FHWA = U.S. Federal Highway Administration, DoT = U.S. Department of Transportation, and NOAA = U.S. National Oceanic & Atmospheric Administration. ** Code: To simplify the data collection process, we present the **R** code needed to scrape and clean these different data sources at: https://caimiao0714.github.io/TrafficSafetyReviewRmarkdown/.

Factors that have been shown in the literature to contribute to motor vehicle crash risk are discussed in detail in Section 4. Here we concentrate on strategies and sensing technologies used to obtain relevant data. From a data acquisition viewpoint, the sensors can be divided into [27]: (a) intra-vehicular sensing platforms, where conditions extracted from the vehicle are captured, and (b) urban sensing platforms, where the sensors are integrated in the road infrastructure. Intra-vehicular sensors can capture driver behavior, vehicle speed, traffic environment, etc. [28], and are widely used in NDS studies. On the other hand, urban sensing platforms are more commonly utilized in case-control studies.We can categorize such platforms into the following three categories: (a) traffic sensing systems (e.g., traffic cameras, inductive loop detectors, infrafred sensors), which can be used to estimate traffic flow, speed, occupancy, and volume [27]; (b) weather sensing systems, which can be used to compute/estimate important factors for both explanatory/predictive (e.g., visibility, rain/snow accumulation, and potential for icy conditions) and prescriptive modeling (e.g., wind direction and speed which are important considerations in hazardous material routing since they are used in predicting the severity of a possible crash through estimating the radius of dispersion of toxic materials); and (c) geometric road descriptors (e.g., number of lanes, speed limit information, longitudinal grade, road shoulder width, and whether the road segment of interest contains a straight, merge, and/or diverge sections), which are typically tagged in geographic information systems (GIS) and can be accessed using popular application programming interfaces (APIs) such as *OpenStreetMaps* [29,30]. A visual summary of predictor variables extracted from urban sensing systems is provided in Figure 3.

## 3. Descriptive Analytic Tools Used for Understanding Crash Data

**Figure 4:**
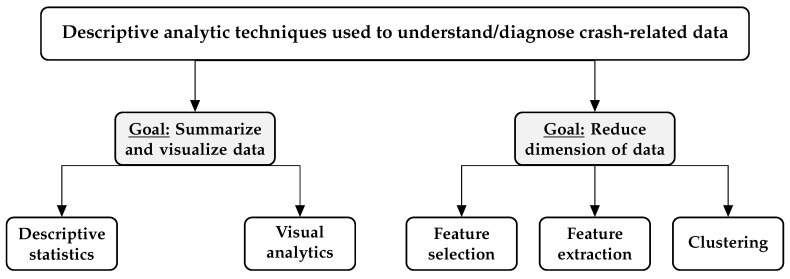
Exploratory data analysis (EDA) goals and their associated techniques/methodological frameworks.

In this section, we review the exploratory data analysis (EDA) techniques used to examine transportation datasets prior to the explanatory/predictive modeling stage. EDA is an especially important pre-processing steps when dealing with large datasets, where predictive modeling and optimization can be computationally intensive. In Figure 4, we depict the two major goals of EDA as well as the methodologies used to achieve these goals. Note that these methods may not be mutually exclusive and can be used to complement each other.

### 3.1. Data Summarization and Visualization

Data summarization include both univariate (e.g., central tendency, dispersion, etc.) and multivariate tools (e.g., correlation). We assume that both predictive and prescriptive modeling researchers are well-versed with these methods, and thus we will not discuss them here (see Washington et al. [31] for a detailed introduction). As a complement to data summarization, data visualization is a succinct approach to understanding trends, patterns, and anomalies in data. In a survey paper on the application of visualization techniques for traffic datasets, Chen et al. [32] categorized visualization approaches based on four data types: (a) temporal data, (b) spatial data, (c) spatiotemporal data, and (d) multivariate data. This framework can be extended to more comprehensive crash modeling studies where traffic, weather and other predictor sets are combined. Table 1 presents an overview of the appropriate/recommended visualization techniques for each data type, with example references from the literature. In the following subsections, we discuss each of these groups in further detail.

#### 3.1.1. Visualization of Time-Oriented Data

Line graphs are the most frequently used visualization technique for time-oriented data, where the *x-*axis represents time and *y-*axis demonstrates transportation-related variable. There are numerous applications of line graphs in traffic/crash visualizations, for example, visualizations of tips per trip and fare per miles-driven among New York City taxi drivers [36], carbon monoxide pollution over the course of the day in London [60], traffic volumes in Beijing, China [33] and Porto, Portugal [34], or the effects of road surface conditions and time of day on traffic volumes [35]. Since line graphs can become visually overwhelming as the number of variables increases. Other time-series based graphs can be considered in this case, such as *ThemeRiver stacked chart* [61], which uses a flowing river metaphor to capture changes in several variables of interest over time. This chart was used by Guo et al. [37] for understanding traffic volume patterns.

When the data are inherently periodic or cyclic, three charts can be applied [32]: radial layout, cluster- and calendar-based (where line graphs are used for showing cluster averages over time, and calendar-based charts are used to show cluster membership per day) [62], and statistically derived charts. Pu et al. [39] used the radial layout chart to depict traffic volumes in different days and times. Tsai et al. [38] showed how the cluster- and calendar-based charts can be effective in understanding traffic flows in the state of Alabama. In their case study, they showed that the data exhibited eight distinct clusters of daily traffic volumes (at hourly intervals within each day). Two of the clusters were somewhat unexpected, where one captured game-day traffic for college football, and the other captured travel patterns around different holidays (including Fourth of July, Thanksgiving, and Christmas). Statistically derived plots (based on time-series analysis techniques) can be used to quantify the periodic/seasonal nature of the data. From a time-series analysis perspective, the data can be decomposed into: (a) seasonal, (b) trend, and/or (c) cyclical components within a season. These components can be visualized, along with the autocorrelation function (ACF) and the partial autocorrelation function (PACF) for the differenced series to provide an understanding of what type of time-series models to use. The reader is referred to [31] for a detailed coverage of time-series modeling applied to transportation data analyses.

#### 3.1.2. Visualization of Spatial and Spatiotemporal Data

Crash datasets provide rich spatial information including the location of vehicles, construction sites, road closures, and crashes. Visualizing them spatially gives insight(s) on the geographical patterns and clusters, which may improve the decisions made when setting up the dataset for predictive/ prescriptive modeling. Chen et al. [32] presented three visualization options (point-based, line-based, and region-based visualizations), which should be selected based on the dataset’s aggregation level.

**Figure 5:**
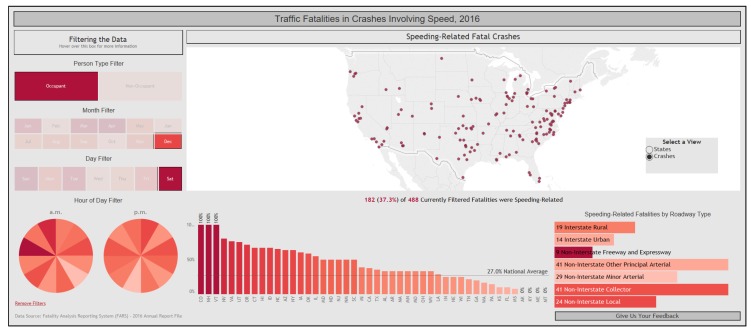
Symbol map showing the location of vehicle occupants killed in speed-related crashes in the US in December, 2016. The dashboard is available at [40].

In point-based visualizations, each symbol on a map represents the position of an object at a given point in time. An example of such a visualization is the motor vehicle fatality symbol map, which is used by NHTSA to depict fatalities [40]. We provide a screenshot of their dashboard in Figure 5, showing the location of vehicle occupants killed in speed-related crashes on Saturdays in December 2016.

Popularized by the ubiquity of modern navigation applications, a line map visualizes travel routes and traffic flow. An example can be found at [42], who presented the trip patterns in Bristol, England. They used the “line width” to encode the number of trips and “color” to encode active travel percents. Given the widespread use of navigation applications, we do not discuss other examples in this review.

Region-based spatial visualizations include three popular visualization techniques. The first is the “proportional symbols map” [43], where the size of a point/symbol in a map is proportional to the number of observations in that location. This can be seen as an extension to the point-based visualization, where the point-position on the map is now used to encode count. The second technique is based on “choropleth maps” [44,45,46], where areas/regions in maps are shaded, colored, or patterned relative to the value of the metric of interest. These maps are common when comparing crash/fatality rates between larger geographic regions (e.g., counties, states, or countries). The third, and least commonly used visualization is the “radial metaphor”. One existing application was provided by Zeng et al. [47], who used a “radial metaphor" chart to visualize interchanging traffic patterns among different regions of a city.

For spatiotemporal visualizations, there are two overarching strategies that can be used. The first strategy is intended for web-based visualizations, where a time effect is added to the map by animation or transition effects. Examples can be found in [50,51]. On the other hand, the second strategy is intended for print and utilizes dedicated visualization methodologies. Space-time-cube (STC) visualizations, are the most commonly utilized approach, where the *x* and *y* axes are used to capture spatial information, while the temporal information is shown on the *z* axis [48]. Applications of such technique include: (a) traffic analysis where the changes in a traffic-related variable of multiple vehicles across time and space is shown by stacking-based STC [52]; and (b) crash analysis where crashes are displayed and tracked based on their spatiotemporal information by an enhanced version of standard STC [49,63]. Despite their perceived utility for showing spatiotemporal patterns in a 2-dimensional screen/paper, we do not recommend this approach since the actual values cannot be easily shown and comparisons depend on one’s ability to estimate the patterns over space and time. Instead, we would recommend the use of either panel visualizations (i.e., trellis/ small multiples), or a tabulated representation of the results to show the time component.

#### 3.1.3. Visualization of High-Dimensional Datasets

For high-dimensional data, visualization requires more data cleaning and curation. On the lower end of the spectrum, Parallel coordinates plots (PCP) and trellis (small multiples of bar charts or scatter plots) are commonly used fast plotting tools and require less data preprocessing. For example, PCP can be applied to visualize the correlation/interaction among several crash descriptors including: cars involved, day/month effects, incident type, and road condition [45,53,55]. Additionally, the trellis plot was used by Cottrill and Thakuriah [54] to visualize variations in the number of crashes by different census tracts. On the upper end of the analytical spectrum, visualizations are preceded with the application of projection methods to reduce the problem’s dimensionality. Examples include: (a) Van Huysduynen et al. [57] where cluster analysis and multidimensional scaling were used to produce a 2-dimensional (2D) plot of the relationship between the different constructs and types of drivers examined in the study; (b) Das et al. [59] who utilized multiple correspondence analysis (MCA) to present a proximity map of key factors contributing to wrong-way driving in a 2D space; (c) Liu et al. [58] where the multivariate time-series data capturing the driver behavior were reduced to a 3D feature space using deep learning techniques, and then visualized using a driving color map.

### 3.2. Dimension Reduction

In the previous subsection, we highlighted how projection methods can be used to reduce the data dimensionality and assist in its visualization. Here, we discuss how dimension reduction techniques can be used to prepare the data for the predictive modeling stage. In general, there are three main goals for dimension reduction: (a) feature selection, where important variables are identified and selected; (b) feature extraction/generation, where the variable set is projected into lower subspace without losing significant information and; and (c) clustering, where similar observations are grouped together. Since researchers could combine these approaches in their analysis, we classified dimension reduction methods according to their goals.

#### 3.2.1. Feature Selection

One of the recommended steps before the use of statistical and machine learning models is to identify and use only the variables/features deemed important for the analysis since this [64]: (a) avoids over-fitting, (b) reduces the computational complexity in the analysis, and (c) leads to better prediction performance. This step is often referred to as variable or feature selection. In the context of crash prediction models, variable selection plays an important role since there are many potential predictors (e.g., traffic, weather, road geometry related variables) which may have effect on the probability of a crash. In addition, in order to capture the spatial and temporal effects of these variables, new variables need to be introduced in the model. For instance, Shi and Abdel-Aty [65] developed a crash prediction model where each traffic-related variable is collected prior to the crash from two upstream and two downstream sensors. This means that the information for each traffic variable is divided across four variables, and that these variables contain some redundant information within them. In such cases, feature/variable selection will improve model performance [66,67,68,69,70]. For the sake of conciseness, hereafter we use the term feature selection to denote feature and variable selection methods.

Feature selection methods can be classified into three groups: filter, wrapper, and embedded methods [71]. In the filter methods, the process of selecting a subset of features is independent from the statistical and machine learning model used, i.e., a subset of features will be selected according to an algorithm (e.g., Pearson correlation or Mutual Information Criterion), and then the selected features will be the inputs to the explanatory/predictive model. Advantages of filter methods include: (a) simplicity, (b) computational efficiency, (c) speed, and (d) reduction of the risk of over-fitting. However, they can ignore the dependency between features and do not guarantee the selection of an optimal set of features [71,72]. In contrast, wrapper methods consider the prediction performance of the classifier (while accounting for the dependencies/interactions between features) and subsets the feature space using heuristic searching algorithms such as genetic algorithms [73] and particle swarm optimization [74]. While they can improve performance when compared to filter methodologies, they are computationally inefficient. In addition, they also do not guarantee optimality and may over-fit [71,72]. To avoid such problems, feature selection is a part of the model training process in embedded approaches, which makes them the preferred approach in many crash risk modeling scenarios. Random forest (RF) was widely used in the literature as a feature selection method and to determine variable importance [69,70,75]. For more information about the feature selection methods and their applications, we refer the reader to Saeys et al. [72], Guyon and Elisseeff [76], Jović et al. [77].

#### 3.2.2. Feature Extraction

Feature extraction methods offer an alternative approach to dimension reduction by projecting input space to a more efficient dimension space. The projection can combine input variables, reduce the problem complexity, and present a useful abstraction of the data [78]. Thus, feature extraction differs from feature selection as the focus is not on dropping unimportant variables, but rather to combine the information across the variables through a mathematical transformation. Principal Component Analysis (PCA) is the most commonly used feature extraction method in the crash prediction literature [79,80,81,82,83,84]. Through an orthogonal transformation, PCA transforms the original variables into a set of linearly uncorrelated variables (i.e., principal components, PCs). Typically, the variation in the data can be explained with a few PCs, which reduces the dimensionality of the problem with minor loss of information. The determination of the number of PCs to retain is often determined through a scree plot or a threshold for the eigenvalues [85]. Since PCA was originally designed for numeric variables that can be linearly combined, there are several extensions to PCA which do not require such assumptions. These include: (a) probabilistic PCA [86], (b) non-linear PCA [78], and (c) kernel-based PCA [87]. These methods have also been implemented extensively in the literature [78].

#### 3.2.3. Clustering

Contrary to feature selection and extraction, clustering is an unsupervised machine learning method that attempts to group observations together with the goals of maximizing the similarity within a cluster (i.e., minimizing distance between observations) and minimizing the similarity between clusters (i.e., maximizing the distance between cluster centers/centroids) [88,89]. Clustering approaches can be divided into: partitioning-based, hierarchical-based, density based, grid-based, and model-based methodologies [88,90].

Crash risk modeling datasets have a number of characteristics that make clustering a viable and useful approach for dimension reduction. For example, if you consider traffic datasets, the goal is typically to understand the impact of traffic conditions on crash likelihood, which is typically achieved through: (a) classifying traffic into different states, and then (b) evaluating the impact of each traffic state (e.g., congested or not congested) on the crash likelihood [10]. Historically, step (a) was achieved through an analysis of traffic flow characteristics (e.g., see [91,92,93]). A limitation of such an approach is that the modeling can be influenced by researchers’ biases and perceptions. Alternatively, one can use an assumption-free, data-driven approach to identify how observations can be clustered. Tsai et al. [38] showed how clustering can be used to identify logical, but hard to model, groupings of the data. Applications of clustering include, but are not limited, to: (a) traffic categorization [38,94,95], (b) identifying accident clusters [96,97,98], and (c) grouping of weather conditions [99]. To demonstrate how an optimal number of clusters ($k^{*}$) can be obtained, we provide a detailed example in the Supplementary Materials where we use k-means clustering and the elbow method to determine the $k^{*}$ clusters for traffic data.

## 4. Explanatory/Predictive Models for Crash Risk

This section focuses on two aspects: the risk factors that affect crash risk and statistical/machine learning models. In the risk factors part, we specifically consider the effects of fatigue, distracted driving, and environmental variables including traffic and weather on traffic safety. For the statistical part, we will review how some of the research that has been done to analyze those factors and build predictive models.

### 4.1. Risk Factors for Traffic Safety

Roshandel et al. [11] discussed five sets of factors that affect crash risk: (a) behavioral characteristics of the driver—e.g., impairment, fatigue, distractions; (b) vehicle condition; (c) traffic conditions—e.g., traffic speed, density and variation in speed between vehicles; (d) geometric characteristics of the road, i.e., type of road, number of lanes, curvature, nearby ramps/intersections, etc.; and (e) weather conditions—e.g., rain, visibility, ice/sleet/snow, etc.

#### 4.1.1. Sleep and Fatigue

Early work on the study of fatigue and the risk of adverse outcomes such as crashes relied on sample surveys of drivers. For example, Crum et al. [100] conducted face-to-face interviews with approximately 500 truck drivers at five rest stops on interstates spread across the United States. The three outcomes were “close calls,” “perception of fatigue,” and “crash involvement.” All of these were based on driver recall from survey responses. They identified three sets of variables that could affect drivers’ fatigue, with self-reported measures. These measures included truck driving environments, economic pressures, and carrier support for safety. Three specific variables, all from the truck driving environment category, were identified as influencing fatigue, including: (a) drive regular or irregular shifts; (b) short or long load wait time; and (c) start the work week tired (or not). Crum et al. [100] ran a regression analysis with these factors as predictors, with each of the responses described above. The first variable (drive regular or irregular shifts) was measured by determining how many six-hour times periods the drivers routinely drove. They found that starting the work week tired was a significant predictor for all three outcome measures described above. Long wait times were positively associated with close calls and self-perception of fatigue. Paradoxically, the number of time periods driven per day was negatively associated with close calls.

In another early study, Crum and Morrow [101] conducted a stratified sample of trucking companies based on their safety record. They selected a sample from each of three strata defined as the bottom quartile (poorest safety performers), the middle two quartiles, and the top quartile (the highest safety performers). After taking a sample of carriers within each stratum they sent seven questionnaires to be filled out by various employees in the company, including the executive, the safety director, two dispatchers and three drivers. They also arranged focus groups within each company. Using the same three sets of variables as in [100] they concluded that the most significant variable in predicting fatigue was “starting the workweek tired.” Other significant factors were “difficulty finding a place to rest” and “shipper and receiver scheduling practices and requirements.”

Garbarino et al. [102] conducted a cross-sectional study of truck drivers in Italy to determine the risk factors for accidents and near misses. Data on sleep apnea, sleep debt, daytime sleepiness, frequency of naps, and frequency of rest breaks, as well as the accident responses were conducted from survey questionnaires and medical exams. They found that obstructive sleep apnea, sleep debt, and excessive daytime sleepiness were positively correlated with accidents; these yielded odds ratios of 2.32, 1.45, and 1.73, respectively. Naps and rest breaks were negatively associated with accidents, having odds ratios of 0.59 and 0.63 respectively. All of these odds ratios had confidence intervals that excluded the null value of 1.0.

With automatic data collection systems that can detect events like accidents, hard-breaks (sudden deceleration caused by braking), lane departures, and others. Mollicone et al. [21] studied hard braking as safety critical events, which are highly correlated with crashes [103]. Their model used a predicted fatigue model of McCauley et al. [104] and McCauley et al. [105] to develop a Poisson regression model having the number of hard brakes as the response. The predictor variables included the predicted fatigue and six variables for the time of day. They found that there is an increasing and concave up relationship between the predicted fatigue and the relative risk of a hard brake.

In a recent study, Stern et al. [106] reviewed the research related to fatigue of commercial motor vehicle drivers. Because of the difficulty of running a controlled experiment by imposing treatments, most research designs are observational studies, that is, they compare the effects of variables that are observed, not imposed. One exception to this is a *randomized encouragement design* where drivers are randomized to receive some sort of incentive to apply some treatment, but are not forced to do so. If an effect is observed, we would conclude that it is due to the incentive, not necessarily to the actual treatment. Many studies use a cohort design or a case-control study. In a cohort design, a number of drivers is identified and studied across time. In a case-control study, a number of cases (e.g., crashes) are identified and are matched with controls; focus is then placed on the differences between the cases and controls. Both cohort studies and case-control studies can be useful in assessing safety.

Recently, Bowden and Ragsdale [107] developed an optimization algorithm for driver scheduling. The algorithm, denoted FAST (Fatigue Avoidance Scheduling Tool) was designed to minimize the trip duration subject to a minimum fatigue level along with other constraints, such as the maximum driving hours under United States law. The algorithm assumes the three process model of alertness (TPMA) developed by Åkerstedt and Folkard [108] and Åkerstedt et al. [109].

#### 4.1.2. Distracted Driving

Other researchers have looked at the effect of distracted driving. The problem of mobile phone usage and distracted driving has been noticed by the World Health Organization [110]. They noted that world-wide use of cell phones has increased by up to 11% in the past 5 to 10 years. Their data suggest that cell phone usage increases the chance of a crash by a factor of four, and this is similar for hand-held phones and hands-free devices. Young et al. [111] noted that at the time, about one fourth of all crashes (trucks and personal vehicles combined) were due at least in part to distractions, particularly mobile phones and navigational systems. They reviewed much of the literature available at the time of their writing. Wilson and Stimpson [112] reviewed trends in distracted driving accidents and noted that deaths due to distracted driving had increased 28% from 2005 to 2008 when the rate was nearly 6000 deaths per year.

Olson et al. [113] studied distracted driving in 203 commercial drivers. The data involved 4452 critical events, such as crashes, near-crashes, and unintentional lane departures, along with 19,888 time periods that involved no special events. The found that 71% of all crashes and 46% of near crashes involved drivers who were engaged in tasks not related to driving. Overall, 60% of critical events occurred while the driver was performing non-driving tasks. Klauer et al. [114] conducted a study in which 42 young drivers (16.3 to 17.0 years of age) who had just received their driver’s license and 109 experienced drivers were studied. Here the unit of measurement is the driver. Equipment, such as accelerometers and cameras, were used to detect distracted motion while driving. They found that distracting events like eating or cell phone dialing or texting led to an increased risk of accident, with odds ratios often exceeding 3.0.

In terms of safety optimization, the choice here is clear. Distracted driving, such as hands-on cell phone use and texting, should not be allowed. From a general public perspective, these have translated into driving laws in many countries as well as have been translated into company policies for many commercial transportation firms. In addition, there are several smart phone-based applications that disables texting while driving and/or encourage safe driving behavior. From a commercial driving perspective, there are wearable technologies (e.g., headsets embedded with sensors that are linked to a smartphone application) that are used by professional drivers that provide voice-alerts when their mirror-check rate deviates from a pre-set standard. This information is also shared with dispatchers to schedule rest-breaks as an intervention. While these smart-phone applications/technologies seem promising, there is not a large body of literature that examines the effectiveness of these interventions.

#### 4.1.3. Weather, Traffic Conditions, and Road Geometry

In Section 4.1.1 and Section 4.1.2, we have discussed driver-related factors. In many cases, the crash likelihood and severity can be impacted by non-driver/external factors. Variables/features capturing weather (e.g., temperature, precipitation, wind speed, humidity, and visibility), traffic conditions (e.g., traffic flow, occupancy, density, and volume), and road geometry (e.g., elevation, curvature, road surface, and the number of lanes) represent the main external factors that impact the crash likelihood and severity [10,11,115]. Note that these factors should not be considered in isolation since their interactions are complex and can significantly change the crash likelihood. Thus, in this subsection, we highlight three relevant studies that have investigated the combined effect of such factors on crash risk.

Ahmed et al. [116] investigated the effect of the interaction between road geometric features, real-time weather parameters, and traffic data on crash likelihood. Using a Bayesian logistic regression framework, the authors developed two models for snowy and dry seasons. Based on their models and case study, their results showed that in, both models, the main effects and at least one interaction term were significant. The authors showed that the crash risk during the snowy season was two times that of the dry season. Furthermore, the authors suggested that the crash risk likelihood may also be influenced by the interaction effects between the snowy, icy, or slushy road surface conditions with road segments involving steep grades.

In another study, Yu et al. [117] conducted their study on a 15-mile segment of the I-70 interstate in Colorado. The authors utilized: (a) 30 Remote Traffic Microwave Sensor (RTMS) sensors to extract real-time traffic data; (b) six weather stations for obtaining real-time weather data; and (c) the Roadway Characteristics Inventory (RCI) for obtaining descriptors of road geometry. Different scenarios were considered in the study based on the season and crash type. The results showed that the adverse weather condition combined with critical roadway conditions (e.g., steep slopes) can increase the crash likelihood significantly. Further, single vehicle (SV) and multiple vehicle (MV) models shared some common significant predictors such as precipitation and average speed. Furthermore, in the SV model, the significant variables were more related to weather conditions and vehicle speed. On the other hand, MV crashes were more affected by traffic-related variables.

Wang et al. [118] studied several of the factors that could lead to high risk traffic conditions. They considered traffic, weather, road geometry, and some behavioral aspects, such as trip generation and social demographics. These variables were taken as the characteristics of the region surrounding the crash, not the individuals involved in the crash. They used a case-control design with a 10:1 ratio of non-crashes to crashes. They used support vector machines (SVM) for variable selection and Bayesian logistic regression for inference. They found that the percentage of home-based work production, which includes commuters, was the only behavioral characteristic that had a significant effect on the risk of accident.

Xu et al. [115] developed crash prediction models at different levels of crash severity. Three levels of crash severity were considered: fatal/incapacitating injury crashes (KA), non-incapacitating/possible injury crashes (BC), and property-damage-only crashes (PDO). Results showed that under different crash severity levels, the effect of environmental variables is different. For example, in the all crashes model (KA, BC and PDO), adverse weather conditions would increase crash risk. However, under the injury crashes model (KA and BC), adverse weather conditions had the opposite effect which indicated that it could possibly reduce the likelihood that a crash would result in injuries and fatalities (possibly due to uncaptured changes in driver behaviors). Note also that the significant traffic-related variables are different in these two models which indicates that the interaction of the external variables would result in different level of crash risk and severity.

### 4.2. Statistical Modeling

Retrospective case-control studies are usually analyzed using logistic regression or other classification models. Since crashes are very rare compared with non-crashes, matching a case (crash) with one or more controls (non-crashes) is considered; this matching is then accounted for in the analysis. In other situations, non-crashes are unmatched; a set of controls is selected to mimic the aggregate of conditions of the crashes. Many studies are unclear about matching and whether (and also how) the matching was taken into account in the analysis. Since non-crashes are much more common than crashes, it is common to take several times as many non-crashes as crashes. Ratios as high as 10:1 are found.

Theofilatos and Yannis [10] surveyed previous research on the relationships between these factors and traffic crashes. Some of the commonalities in the conclusions were that safety was a nonlinear function of traffic flow, speed limits were a factor, and precipitation was related to accident frequency, although the effect on severity is unclear. Roshandel et al. [11] conducted a review and meta analysis of previous traffic safety studies and found that four variables are likely contributors to accident likelihood. These include speed variation around the crash site (odds ratio = 1.226), speed difference (odds ratio = 1.032), average traffic volume (odds ratio = 1.001) and average speed (odds ratio = 0.952).

Shi and Abdel-Aty [65] used a matched control design to study rear end crashes. They matched 243 crashes with 962 non-crashes, a ratio of about 4:1. They used a random forest for variable selection, and then used a Bayesian approach for logistic regression. They found that peak hour, high volume upstream (from the accident), low speed downstream, and high congestion index downstream were significant factors for rear end crashes. Pande and Abdel-Aty [119] also studied exclusively rear end crashes in an unmatched case-control study. They found 2179 rear-end crashes, but only 1620 with full data, in a period of five years and selected a random sample of 150,000 of the roughly 363 million possibilities for the controls. They used classification and regression trees (CART) to discriminate low and high risk situations. Their approach could classify a situation as high-risk about 75% of cases where there was an accident, with approximately a 33% positive rate. Since crashes were rare events, one can conclude that their false positive rate was ≈ 33%.

In a later study, Pande et al. [120] studied rear end crashes in a case-control study. They used a 5:1 ratio of non-crashes to crashes and used a random forest for variable selection and a multilevel perception neural network for inference. They found that occupancy downstream and average speed upstream were significant.

Theofilatos et al. [121] studied traffic safety on a multi-lane belt-line highway in Athens, Greece, where there were 17 crashes and 91,118 non-crashes. In one model they use all data, and in another model they use a random sample of the non-crashes. They assume a logistic regression model and in one model they use a penalized maximum likelihood approach, called the Firth method, which uses all of the data. In another approach, they use a bias correction method to estimate parameters in the logistic regression, and for this they use a subset of the data. They find that average speed has a negative effect on crashes. The proportion of trucks on the road was considered but not found to be significant.

Lin et al. [122] studied traffic safety on a corridor of Interstate 64 in Virginia, USA. Their study used a matched case-control design. They propose a frequent pattern (FP) tree which they use for variable selection. For inference on which variables are significant they use a *k* nearest neighbors algorithm and a Bayesian network. They conclude that the “accident risk prediction models based on FP tree variable selection outperform the models based on all variables …” They also suggest using 10-minute intervals is more efficient than 5-minute intervals. Finally, they conclude that the Bayesian network model works well, yielding a false alarm rate of 0.38 and a sensitivity of 0.61.

Sun and Sun [123] used a matched case-control design with a ratio of 5:1 to implement a Markov model involving the traffic states upstream and downstream. For example, if one upstream and one downstream segment is considered, then an expressway segment may be in the state FF (free flow upstream and free flow downstream); this leads to a four-state Markov chain. They also consider two upstream and two downstream conditions, leading to a nine-state Markov chain. The transition probabilities were estimated using a dynamic Bayesian network model. Their model with nine states had a crash accuracy of 0.764 with a false alarm rate of 0.237. In addition to their work on the Bayesian network, they found an interesting nonlinear relationship between speed and risk, which they show in the second figure of their paper.

The effect of weaving, that is, traffic entering the expressway and merging while other traffic is exiting, was studied by Wang et al. [124] in a case-control study of 125 crashes and 1250 non-crashes, a 10:1 ratio. They applied a multilevel Bayesian logistic regression model with weaving segments (that is, sections of the expressway where entering and exiting traffic had to merge) as random effects. These random effects were incorporated into the model as random intercepts. They found that the speed at the beginning of the weaving segment, difference in speed between the beginning and end, and the log of traffic volume were significant effects in these weaving segments. Wang et al. [125] approached the traffic safety problem from two perspectives. One involved the crash frequency. This took as the sampling unit a section of the expressway and the number $Y_{i}$ of accidents as the response. The other approach applied the usual logistic regression, taking the sampling unit as an expressway/time period slice and the indicator variable $y_{ij}$ which is 1 for crash and 0 for a non-crash. The first approach leads to Poisson regression and the second approach leads to the usual logistic regression. The innovative contribution of their method is to combine, or integrate, these two models. This effectively uses two sources of data. Their integrated model includes the Poisson rate in the logistic regression model, yielding a multi-level model. They find that the integrated model performs better yielding a higher receiver operating characteristic (ROC) curve.

There are a lot of aspects of crash prediction models that can be studied, including model setting, specification, and validation, but those are beyond the scope of this review. Details of statistical models can be found in previously published reviews by Lord and Mannering [126], Mannering and Bhat [127], Abdulhafedh et al. [128], Ambros et al. [129], Yannis et al. [130].

## 5. Conclusions

Given the tremendous loss of life and property directly attributed to motor vehicle incidents on one hand, and significant advances in relevant data availability on the other, it is natural that data analytics is viewed as having great potential for contributing to solving these problems. A successful effort in this direction necessarily has to rely on a combination of data collection, descriptive analytics, predictive/explanatory modeling, and optimization. At the same time, each piece separately can be a significantly nontrivial problem on its own. Hence, development of a mature data-driven decision support tool incorporating all of these stages “from scratch” is probably beyond the scope or ability of any single researcher. This is especially true since there is not a conscious effort in pulling all of these areas together with the goal of informing practical decision-making. The most significant gap that we have identified, is in the translation of outcomes/insights from predictive/explanatory models (which aim to help us better understand and quantify crash risk) into prescriptive optimization models (which aim to inform route/path selection, driver assignment, etc.). Perhaps, a partial underlying reason is the absence of readily available convenient data sources and/or data processing tools.

In this review, we highlighted a promising opportunity to develop advanced analytical methods for safety-enabled transportation. The following areas represent the main avenues for progress (ordered according to the sections in this review):

(A) The availability of historical, real-time and forecasted weather and traffic data, as well as the potential to collect driver performance data, means that the accessibility of data is no longer a major factor preventing progress in this area. However, a lack of a unified repository and the reluctance of sharing code/models by our research community leads to a fairly high overhead cost of developing such models (since every researcher has to develop many data collection techniques from scratch);
(B) Descriptive analytics tools are widely used in the preprocessing of driving-related data. Since the applicability of a particular preprocessing technique (e.g., visualization and clustering) often depends on the specific problem, the challenge here is to determine which method is the most suitable. Sharing best practices by creating reproducible documents (e.g., R Markdown and Jupyter notebook) represents one avenue for making the process more efficient for researchers and practitioners alike.
(C) Statistical methods for risk evaluation are well-researched and consider a wide range of factors. At the same time, it must be noted that (in some cases) these studies follow a similar pattern of a case-controlled study based on a single road segment data. In our view, there is an opportunity for a statistical analysis of a larger scale since:
  (i) real-time or near-real-time data are more widely available now;
  (ii) the computational advancements in the recent years can allow for parallelizing/ computing risk across the entire road network or at the very least for all major highways and interstates;
  (iii) the insights from these relatively small road segments may not be generalizable to the entire road network; and
  (iv) it is unclear how drivers (regular commuters or commercial) can utilize these insights to make more informed decisions about their time-of-travel, path and/or route selection.

In our estimation, a more comprehensive/interdisciplinary approach to crash risk modeling is needed. The research questions should not be limited to only better understand the factors contributing to crash risk, but to also consider how the output from the research can be utilized by commuters and commercial drivers. This is especially important since, despite the technological advancements in sensing technologies and development of public policies that tackle distracted driving/cell phone usage, the rate and counts of motor vehicle injuries and fatalities have remained alarmingly high.

## Figures and Tables

**Table 1:** Categorizing visualization techniques for transportation data, adapted from Chen et al. [32].

Variable Type(Main Group)	Subgroup	Visualization Techniques	Examples
**Time-series data**	*Linear time*	Line and stacked graphs	[33,34,35,36,37,38]
	*Periodic time*	Radial layout and cluster-and-calendar based visualization	[38,39]
**Spatial**	*Point-based*	Symbol maps	[40]
	*Line-based*	Line maps, edge bundling, and kernel density estimation charts (KDE)	[41,42]
	*Region-based*	Radial metaphor charts, choropleth, proportional symbol maps, and heat maps	[43,44,45,46,47]
**Spatiotemporal**	-	Space-Time-Cube (STC), animated maps, GeoTime, and stacking-based STC	[48,49,50,51,52]
**Multiple properties**	-	Parallel coordinates plot, trellis plot, and multidimensional scaling	[45,53,54,55,56,57,58,59]

## Supplementary Materials

In an effort to bridge the gap between the crash prediction literature and the hazmat/optimization literature, we have made all our source code used for (a) scraping crash-related data, (b) preprocessing of such-data; (c) descriptive analytics (i.e., visualizing traffic/weather/crash data and/or clustering); and (d) explanatory modeling available on a GitHub repository https://github.com/caimiao0714/TrafficSafetyReviewRmarkdown. To facilitate the consumption of this code, we host a website showcasing how the code can be used and depicting some of its results. The website was constructed using an R Markdown file [131], which is stored on the following GitHub Page https://caimiao0714.github.io/TrafficSafetyReviewRmarkdown/. We hope that the Supplementary Materials provided in this manuscript help promote “open data science” practices in our research community.

## Abbreviations

The following abbreviations are used in this manuscript:

AADT	Annual Average Daily Traffic
DoT	Department of Transportation
FHWA	Federal Highway Administration
FMCSA	Federal Motor Carrier Safety Administration
NDS	Naturalistic Driving Study
NHTSA	National Highway Traffic Safety Administration
NOAA	National Oceanic & Atmospheric Administration
VT	Virginia Tech
